# Seroprevalence Dynamics of European Bat *Lyssavirus* Type 1 in a Multispecies Bat Colony

**DOI:** 10.3390/v6093386

**Published:** 2014-09-04

**Authors:** Marc López-Roig, Hervé Bourhy, Rachel Lavenir, Jordi Serra-Cobo

**Affiliations:** 1 Departament de Biologia Animal, Facultat de Biologia, Universitat de Barcelona, Barcelona, 08028, Spain; 2 Institut Pasteur, Unité Dynamique des Lyssavirus et Adaptation à l’Hôte, WHO Collaborative Centre for Reference and Research on Rabies, Paris, 75724, France; E-Mails: hbourhy@pasteur.fr (H.B.); rachel.lavenir@pasteur.fr (R.L.); 3 IRBIO and Departament de Biologia Animal, Facultat de Biologia, Universitat de Barcelona, Barcelona, 08028, Spain; E-Mail: serracobo@areambiental.com

**Keywords:** bats, EBLV-1, Iberian Peninsula, *Lyssavirus*, prevalence, serology, temporal variation

## Abstract

We report an active surveillance study of the occurrence of specific antibodies to European Bat *Lyssavirus* Type 1 (EBLV-1) in bat species, scarcely studied hitherto, that share the same refuge. From 2004 to 2012, 406 sera were obtained from nine bat species. Blood samples were subjected to a modified fluorescent antibody virus neutralization test to determine the antibody titer. EBLV-1-neutralizing antibodies were detected in six of the nine species analyzed (*Pipistrellus pipistrellus*, *P. kuhlii*, *Hypsugo savii*, *Plecotus austriacus*, *Eptesicus serotinus* and *Tadarida teniotis*). Among all bats sampled, female seroprevalence (20.21%, 95% CI: 14.78%–26.57%) was not significantly higher than the seroprevalence in males (15.02%, 95% CI: 10.51%–20.54%). The results showed that the inter-annual variation in the number of seropositive bats in *T. teniotis* and *P. austriacus* showed a peak in 2007 (>70% of EBLV-1 prevalence). However, significant differences were observed in the temporal patterns of the seroprevalence modeling of *T. teniotis* and *P. austriacus*. The behavioral ecology of these species involved could explain the different annual fluctuations in EBLV-1 seroprevalence.

## 1. Introduction

Wildlife plays a key role in emerging infectious diseases by providing a “zoonotic pool” from which pathogens may emerge [1]. Zoonotic pathogens represent approximately 60% of all pathogens able to infect humans [2]. In recent years, bats have been implicated in numerous emerging infectious disease events and have been recognized as important reservoir hosts for viruses that can cross the species barrier to infect humans and other domestic and wild mammals [3]. The role of bats in viral diseases is well established, particularly their role as hosts for lyssaviruses, coronaviruses, flaviviruses, astroviruses and adenoviruses [3,4,5]. Bats have several unique features that may maximize their effectiveness as reservoir hosts for viruses. Bats are the second largest order of mammals. Currently, there are about 1200 recognized bat species worldwide, accounting for approximately 21% of all mammalian species. Bats have the potential to rapidly and widely spread viruses (having a high mobility, they are the only mammals capable of flight). They have a long lifespan and a high survival rate, and many bat species have a gregarious behavior. Bats can fly long distances between their summer and overwintering sites, permitting the exchange of viruses between conspecifics or bats of other species, *i.e.*, in France, rabies virus infections have been associated with the migratory routes of Nathusius’ pipistrelle (*Pipistrellus nathusii*) bats [6]. Persistent viral infections occurring among long-lived bats, coupled with their often gregarious roosting behavior, could greatly increase the potential for intra- and inter-species transmission of viruses [7], especially in summer and winter periods. Seasonality in temperate zone bats includes birthing periods, migration, gregarious behavior and torpor. Each of these strategies may affect population density, contact rates and immune response, thus leading to spatiotemporal variation in infection dynamics [8,9].

Numerous bat species have been found to be infected by lyssaviruses [10]. Bats serve as reservoirs of 13 of the 15 lyssavirus species described (the only lyssavirus species that have not been isolated from bats, to date, are Mokola virus and Ikoma virus). Furthermore, recently described lyssavirus species enlarged the genetic diversity of lyssaviruses found in bats [11,12,13], suggesting that the lyssaviruses originated in these mammals and progressively diverged from a common ancestor [14,15]. In Europe, four of the lyssavirus species recognized, European bat *Lyssavirus* Types 1 and 2 (EBLV-1 and EBLV-2, respectively), Bokeloh bat *Lyssavirus* (BBLV), the West Caucasian bat Virus (WCBV) and one tentative species, Lleida bat *Lyssavirus*, circulate among several bat species [12,16,17]. EBLV-1 is widely distributed throughout Europe, and two variants have distinct distributions and evolution histories: one is EBLV-1a, which has an east–west distribution from Russia to France, with very little genetic variation; and the other is EBLV-1b, which exhibits a south–north distribution and far more genetic diversity [18].

Different studies showed that lyssavirus dynamics exhibits a strong seasonal pattern [8] and that the breeding period could favor the infection of bats [19,20,21]. Many bat species roost in very large and dense maternity colonies. This dense clustering of individuals can provide large opportunities for viral exchange in bat colonies [10]. Previous studies have observed a higher seroprevalence in multispecies colonies compared to monospecific colonies, suggesting that interspecific virus transmission plays an important role in EBLV-1 dynamics [22]. However, in some cases, infection cycles may be maintained among specific host species and transmission may be minimal among sympatric bats [9]. Furthermore, differences in the ecological behavior of species (e.g., migration, torpor) can drive different bat infection dynamics. In this sense, a higher number of species might not only increase the rates of contact between bat groups, but could also facilitate virus entry or spread through the higher mobility of individuals among colonies, especially if there are migratory species involved [22].

Few studies have addressed the inter-annual dynamics of lyssavirus among bat multispecies that are roosting in the same refuge, despite these studies giving a better understanding of the dynamics of bat lyssaviruses. Our previous investigations have analyzed the temporal dynamics of lyssavirus in one bat species (*Myotis myotis*) roosting in two colonies [23,24]. The present report is based on a long-term (nine years) longitudinal study of the prevalence of EBLV-1 neutralizing antibodies and provides the first report on the inter-annual dynamics of EBLV-1 in *P. austriacus* and *T. teniotis*, both being bat species scarcely studied hitherto. We chose this locality, because we found three species (*P. Pipistrellus*, *P. austriacus* and *T. teniotis*) that were EBLV-1 RNA-positive by nested Reverse Transcriptase-Polymerase Chain Reaction in the first year of study [22]. Our specific goals were: (i) to provide information about EBLV-1 seroprevalence in the wild bat community where several European bat species share the same refuge; and (ii) to compare the temporal patterns of seroprevalence mainly in two less-studied bat species that, moreover, exhibit different ecological strategies.

## 2. Experimental Materials and Methods

### 2.1. Study Area

**Figure 1:**
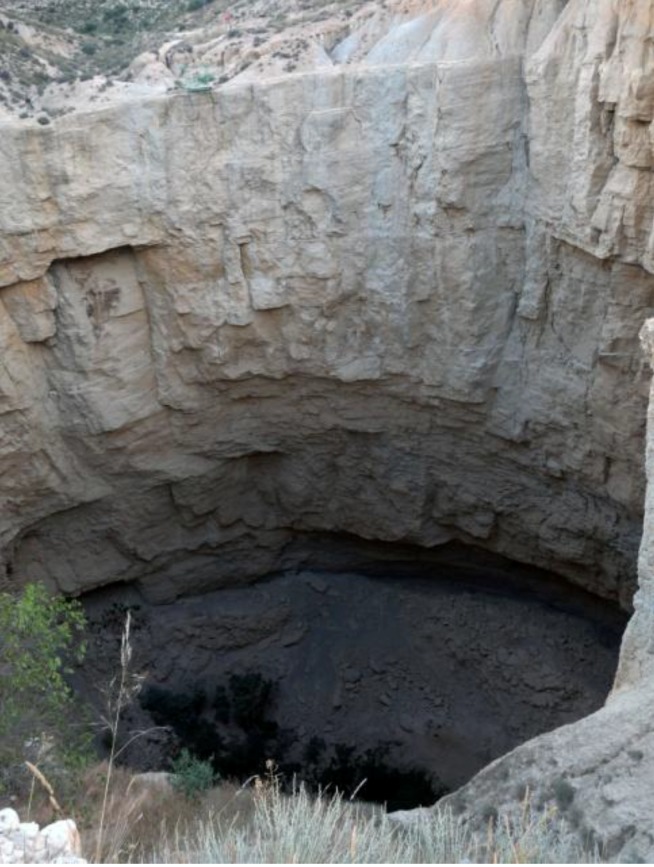
San Pedro de los Griegos pothole.

This study was carried out at the San Pedro de los Griegos pothole (41°1' N, 0°38' E; elevation: 550 m), situated 5 km from Oliete village (Teruel Province). The cavity is an enormous hole with an entrance of 65 × 75 m and a 108-m maximum depth. Crevices in the walls are optimal roost sites for many birds and bat species. However, the pothole is totally illuminated and shows a large lagoon inside (Figure 1).

Around the cavity, the vegetation is dominated by a mix of low growing *Stipa* sp., *Brachypodium retusum*, *Rosmarinus officinalis* and *Thymus vulgaris*. Local weather is characterized by continental climate with a mean annual temperature of 14.60 °C and a mean annual precipitation of 278 mm (mainly in spring). However, mean daily temperature is over 20 °C between June and August (with 15.70 °C and 32.13 °C as the mean minimum and maximum temperatures, respectively). The permanent availability of water and nutrients, the dampening of hard external climatic conditions and the suitability of the habitat for the reproduction of various vertebrate species make the San Pedro pothole a site of unprecedented high biodiversity in Europe [25].

### 2.2. Data Collection

Bats were captured in summer (from June to July) over a 9-year period (2004–2012). Mist nets were employed to capture bats at sunset when emerging from the pothole to forage. All bats were identified to species based on published identification keys of the bats of Europe [26]. Individuals were sexed, and the reproductive status of adult females was classified as pregnant or lactating, based on palpation of the abdomen and nipple condition [27].

Blood samples were obtained by a small puncture made in the median artery. The amount of blood sampled varied from 0.2 mL to 0.5 mL, according to the size of the animal. Pressure with a sterilized absorbent hemostatic sponge impregnated with gelatin was applied to prevent bleeding and facilitate healing. The bats were given 10% glucose water to drink to prevent dehydration and to provide rapidly assimilated compounds for energy. Once bleeding ceased, the bat was released. Vials containing blood were stored at 4 °C for a few hours. Samples were centrifuged for 20 minutes at 9660× *g*, and the serum was extracted with a micropipette. Serum samples and blood pellets were stored at –20 °C before analysis.

All animals were handled in strict accordance with good animal practices, as defined by current European legislation. Bat capture and blood sampling were authorized by permit from the Spanish Regional Committee for Scientific Capture.

### 2.3. Detection of EBLV-1 Neutralizing Antibodies

The technique used to detect EBLV-1 neutralizing antibodies is an adaptation of the Rapid Fluorescent Focus Inhibition Test (RFFIT) [23,28]. A constant dose of a previously titrated (calibrated to give 80% fluorescent foci-infected cells), cell culture-adapted EBLV-1 challenge virus (8918 FRA) was incubated with 3-fold dilutions of the sera to be labelled. After incubation of the serum-virus mixtures, a suspension of BSR cells (a clone of BHK 21 cells) was added. After 24 hours incubation, the cell monolayer was acetone-fixed and labelled with a fluoresceinated anti-nucleocapsid antibody (Bio-Rad, Marnes-la-Coquette, France). The optimal challenge dose (the dilution giving 80% infected cells for each virus production) was calculated. Titers are presented as an arithmetic mean of two independent repetitions. Serum samples with antibody titers <27 are considered negative for EBLV-1-neutralizing antibodies. This cut-off value is similar to that applied in other studies [23,24,28,29].

### 2.4. Statistical Analyses

To study the variation in EBLV-1-antibody prevalence, we conducted two analyses: first, three explanatory variables (sex, species and year) were first screened using a univariate analysis and a chi-square test to check for statistically significant associations with serological status (0: negative; 1: positive). In the second analysis, we used a generalized additive model (GAM) to study the temporal patterns of EBLV-1-antibody prevalence in only two species (*P. austriacus* and *T. teniotis*). More specifically, we used a generalized additive model with the binomial error distribution, where the seroprevalence was the response variable and sex, species and year (2004–2012) were the explanatory variables. The “year” variable was modeled as a covariate fitted with penalized cubic regression splines and sex and species as a fixed categorical factor. To avoid over-fitting and to retain more easily interpretable relationships in the GAM smoothing function, an upper limit of 4 degrees of freedom was set for the year variable when fitting the models. We used an information-theoretic procedure and the Akaike information criterion corrected for small sample sizes (AICc) to compare models [30]. Modeling was performed using the “lme4” and ‘‘mgcv’’ packages in the R program v. 2.14 [31].

## 3. Results

We report the results of the prevalence of specific EBLV-1 neutralizing antibody analysis from the 2004–2012 period in nine bat species roosting in the same refuge. Five of these species (*Eptesicus serotinus*, *P. kuhlii*, *P. pygmaeus*, *Myotis myotis* and *M. daubentonii*) were captured sporadically (sample size <10 individuals during the whole study period), while the rest of the species sampled (*P. pipistrellus*, *Hypsugo savii*, *Plecotus austriacus* and *Tadarida teniotis*) were captured often. The larger samples (>100 individuals) were obtained in *P. austriacus* and *T. teniotis*, because they form large colonies in this cavity. *T. teniotis* form a colony of several hundred individuals. The colony of *P. austriacus* is smaller and consists of 150 individuals, approximately [32].

We observed pregnant females in all bat species, except in *E. serotinus*, *P. pygmaeus* and *M. myotis*, where females were never captured, indicating that this cavity is a breeding roost for the rest of the species found. Males were also captured during the breeding period, indicating that males, either as solitary individuals or forming part of the maternity colonies (e.g., *P. austriacus*), are present during the breeding period in the cave.

### 3.1. Presence of EBLV-1 Antibodies

Among the 406 sera obtained, 71 (17.49%) were positive for EBLV-1-neutralizing antibodies. EBLV-1 antibodies were detected in 6 (66.67%) of the nine species analyzed (*P. pipistrellus*, *P. kuhlii*, *H. savii*, *P. austriacus*, *E. serotinus* and *T. teniotis*) (Table 1). No significant differences in EBLV-1 seroprevalence were detected among seropositive bat species (χ^2^ = 1.67, df = 5, *p* = 0.89). The highest seroprevalence was observed in *H. savii*. We did not find any difference in EBLV-1 seroprevalence between females (20.21%, 95% CI: 14.78%–26.57%) and males (15.02%, 95% CI: 10.51%–20.54%) (χ^2 ^= 1.88, df = 1, *p* = 0.17) when all species were analyzed together and when only bat species with a large sample size—*P. austriacus* and *T. teniotis*—were considered (Table 1).

**Table 1:** The serological results of European Bat *Lyssavirus* Type 1 (EBLV-1) neutralizing antibodies analyzed by all bat species captured in the San Pedro de los Griegos pothole (2004–2012).

Species	Females			Males			Total		
	n	n+	% (95 CI)	n	n+	% (95 CI)	n	n+	% (95 CI)
*E. serotinus*	nd	nd	nd	9	1	11.11 (0.3–48.2)	9	1	11.11 (0.3–48.2)
*H. savii*	7	0	0	15	5	33.33 (11.8–61.6)	22	5	22.73 (7.8–45.4)
*M.daubentonii*	1	0	0	1	0	0	2	0	0
*M. myotis*	nd	nd	nd	1	0	0	1	0	0
*P. austriacus*	76	13	17.10 (9.4–27.5)	56	8	14.28 (6.4–26.2)	132	21	15.91 (10.1–23.3)
*P. kuhlii*	6	1	16.67 (0.4–64.1)	2	0	0	8	1	12.50 (0.3–52.6)
*P. pipistrellus*	9	2	20.22 (2.8–60.0)	19	2	10.53 (1.3 –33.1)	28	4	14.28 (4.0–32.7)
*P. pygmaeus*	nd	nd	nd	2	0	0	2	0	0
*T. teniotis*	94	23	24.47 (16.2–34.4)	108	16	14.81 (8.7–22.9)	202	39	19.31 (14.1–25.4)
Total	193	39	20.21 (14.8–26.6)	213	32	15.02 (10.5–20.5)	406	71	17.49 (13.9–21.5)

n: number of individuals analyzed; n+: number of seropositive bats; CI: 95% confidence intervals; nd: no data.

Capture-mark-recapture of some bats during the study period allowed the tracking of temporal changes in EBLV-1 seroneutralization titers. Seven *P. austriacus* were captured and analyzed almost two times at intervals of one or several years. Four of these seven bats showed positive antibody titers, becoming negative in the following recapture sessions after some years, indicating that these bats survive at least several years after their seroconversion (Table 2).

**Table 2:** Individual serological follow-up in captured-mark-recaptured *P. austriacus*.

Sex	Id	2004	2005	2006	2007	2008	2009	2010	2011	2012
Females	1	0	ns	ns	ns	ns	0	0	ns	52
	2	ns	0	ns	ns	56	ns	43	ns	ns
	3	ns	0	ns	56	ns	ns	ns	ns	ns
	4	ns	ns	ns	ns	35	ns	ns	0	ns
	5	ns	ns	ns	58	0	ns	ns	ns	ns
	6	ns	ns	ns	147	0	ns	ns	ns	ns
Males	7	ns	ns	35	48	ns	ns	ns	0	ns
	8	ns	ns	ns	nd	53	ns	ns	ns	ns
	9	ns	ns	nd	49	ns	ns	ns	ns	ns

Id: identification number of individuals; ns: not sampled; nd: no serological data.

### 3.2. Temporal Variation of EBLV-1 Antibodies

**Figure 2:**
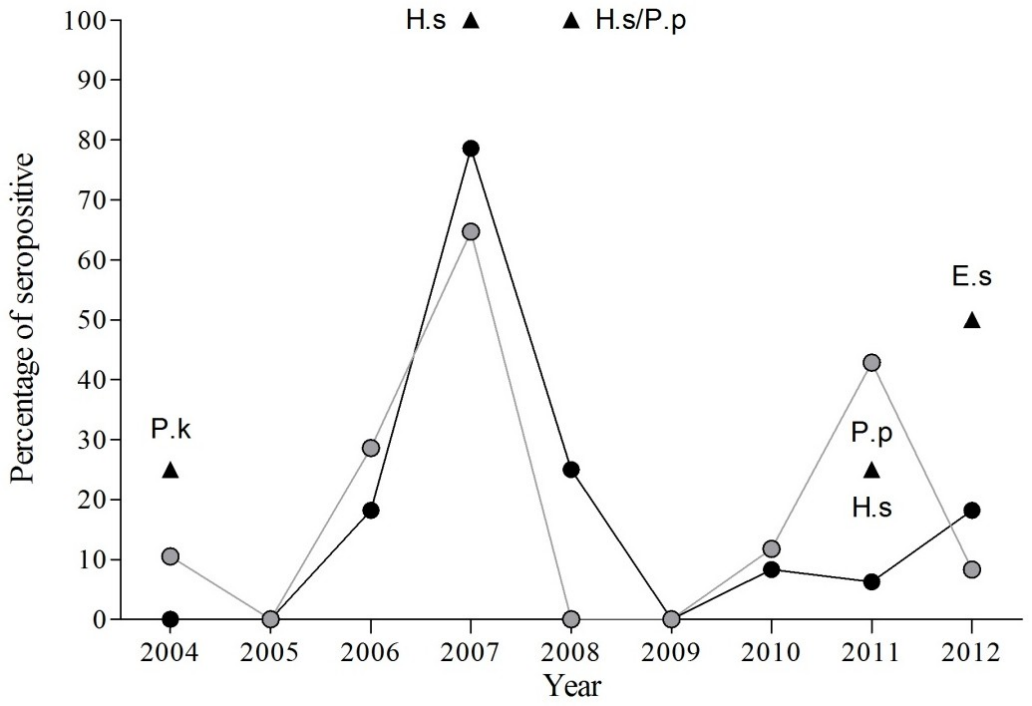
Evolution of percentage of EBLV-1 seropositive bats by species from 2004 to 2012. Black circles for *P. austriacus*, grey circles for *T. teniotis* and black triangles for other species (*E. serotinus*, E.s; *H. savii*, H.s, *P. kuhlii*, P.k; and *P. pipistrellus*, P.p).

The results obtained from 2004–2012 indicate significant inter-annual variations in the percentage of seropositive bats within the study colony (χ^2^ = 94.01, df = 8, *p* < 0.001), with highest seroprevalence in 2007 (70.59%). Only in two years (2005 and 2009) were seropositive bats not detected (Figure 2, Table 3, Table 4 and Table 5).

**Table 3:** The number of bat samples analyzed during the nine-year period.

Years	Females			Males			Total		
	n	n+	% (95 CI)	n	n+	% (95 CI)	n	n+	% (95 CI)
2004	55	6	10.91 (4.1–22.2)	55	1	1.82 (0.0–9.7)	110	7	6.36 (2.6–12.7)
2005	7	0	0	15	0	0	22	0	0
2006	30	9	30.00 (14.7–49.4)	20	3	15.00 (3.2–37.9)	50	12	24.00 (13.1–38.2)
2007	18	14	77.78 (52.4–93.6)	16	10	62.50 (35.4–84.8)	34	24	70.59 (52.5–84.9)
2008	13	3	23.08 (5.0–53.8)	30	6	20.00 (7.7–38.6)	43	9	20.93 (10.0–36.0)
2009	17	0	0	24	0	0	41	0	0
2010	16	1	6.25 (0.1–30.2)	14	2	14.29 (1.8–42.8)	30	3	10.00 (2.1–26.5)
2011	26	4	15.38 (4.4–34.9)	23	8	34.78 (16.4–57.3)	49	12	24.49 (13.3–38.9)
2012	11	2	18.18 (2.3–51.8)	16	2	12.50 (1.5–38.3)	27	4	14.81 (4.2–33.7)

n: number of individuals analyzed; n+: number of seropositive bats; CI: 95% confidence intervals; nd: no data.

**Table 4:** The number of bat samples analyzed, by bat species and year.

Years	*E. serotinus*			*P. kuhlii*			*H. savii*		
	n	n+	% (95 CI)	n	n+	% (95 CI)	n	n+	% (95 CI)
2004	5	0	0	4	1	25.00 (0.6–80.6)	3	0	0
2005	nd	nd	nd	1	0	0	1	0	0
2006	1	0	0	nd	nd	nd	1	0	0
2007	nd	nd	nd	nd	nd	nd	2	2	100.00 (22.4–100.0)
2008	nd	nd	nd	nd	nd	nd	2	2	100.00 (22.4–100.0)
2009	nd	nd	nd	nd	nd	nd	9	0	0
2010	1	0	0	nd	nd	nd	nd	nd	nd
2011	nd	nd	nd	3	0	0	4	1	25.00 (0.6–80.6)
2012	2	1	50.00 (1.3–98.7)	nd	nd	nd	nd	nd	nd

**Table 5:** The number of bat samples analyzed, by bat species and year.

Years	*P. pipistrellus*			*P. austriacus*			*T. teniotis*		
	n	n+	% (95 CI)	n	n+	% (95 CI)	n	n+	% (95 CI)
2004	7	0	0	34	0	0	57	6	10.53 (4.0–21.5)
2005	2	0	0	13	0	0	5	0	0
2006	2	0	0	11	2	18.18 (2.3–51.8)	35	10	28.57 (14.6–46.3)
2007	1	0	0	14	11	78.57 (49.2–95.3)	17	11	64.71 (38.3–85.8)
2008	3	3	100.00 (36.8–100.0)	16	4	25.00 (7.3–52.4)	22	0	0
2009	7	0	0	5	0	0	16	0	0
2010	nd	nd	nd	12	1	8.33 (0.2–38.5)	17	2	11.76 (1.5–36.4)
2011	4	1	25.00 (0.6–80.6)	16	1	6.25 (0.2–30.2)	21	9	42.86 (21.8–66.0)
2012	2	0	0	11	2	18.18 (2.3–51.8)	12	1	8.33 (0.2–38.5)

n: number of individuals analyzed; n+: number of seropositive bats; nd: no data; CI: 95% confidence intervals; nd: no data.

**Figure 3:**
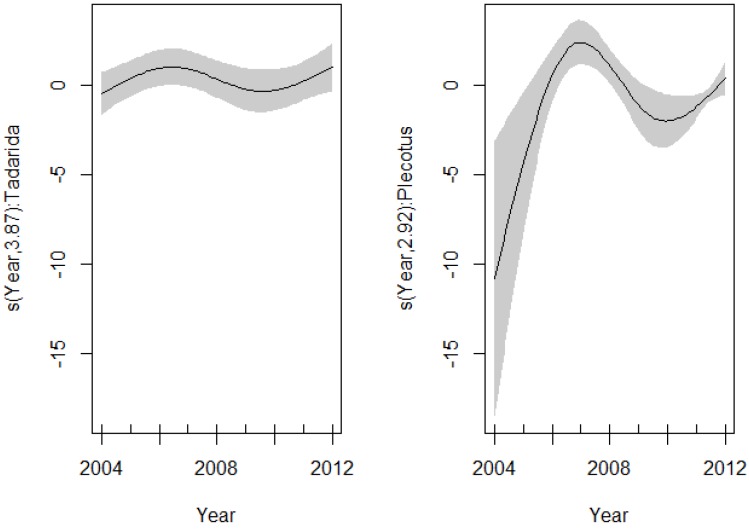
Spline fit (solid line) with 95% confidence interval (dashed lines) of the variability in the EBLV-1 seroprevalence as a function of years (GAM: EBLV-1-antibody prevalence ~ intercept + s(year, by = *P. austriacus*) + s(year, by = *T. teniotis*)). (**Left**) *T. teniotis*; (**right**) *P. austriacus*.

Models that incorporate sex and species variables were not significantly different from the model without these variables (ΔAICc < 2) (Table 6). The best model showed a significant different nonlinear pattern in the EBLV-1 seroprevalence along *P. austriacus* and *T. teniotis*. The effect of year fitted with the spline was highly significant for two species (*P. austriacus*: df = 2.92, *p* < 0.001 and *T. teniotis*: df = 3.87, *p* = 0.026), suggesting a different inter-annual pattern among these species (Figure 3, Table 6).

**Table 6:** Model building results for the generalized additive models (GAM) relating EBLV-1-antibody prevalence and explanatory variables.

GAM model expression	AICc	ΔAICc
1- seroprevalence ~ s(year,by = *P. austriacus*) + s(year,by = *T. teniotis*)	273.22	0.00
2- seroprevalence ~ sex + s(year)	286.49	13.27
3- seroprevalence ~ s(year)	286.90	13.68
4- seroprevalence ~ sex + species + s(year)	287.75	14.53
5- seroprevalence ~ species + s(year)	288.39	15.17
6- seroprevalence ~ sex × species + s(year)	289.53	16.31

## 4. Discussion

Although no positive sera were detected in three bat species (*M. myotis*, *M. daubentonii* and *P. pygmaeus*), this result is probably due to the very low sample size. The high percentage (67%) of seropositive species found and the lack of significant differences in EBLV-1 seroprevalence among seropositive species suggest that most of the bat species can be exposed to EBLV-1 in this pothole although most of these species are not considered as lyssavirus reservoirs by previous studies [12,13,16,33].

Previous studies have shown higher prevalence in females than in males [33,34]. This difference may be due to the gregarious behavior of female bats in summer (nursing colonies are composed almost exclusively of adult females). In these colonies, virus transmission may be favored by high contact rates during social grooming, nursing or olfactory or lingual contact with body fluids. Reproductive activity may also play a role in virus transmission [19], because an increased susceptibility to infectious disease during pregnancy and lactation has been demonstrated in bats [34] and other mammals [35]. However, we report in this study no sex differences of EBLV-1 seroprevalence. The presence of males in this cavity during summer could indicate that males also are present in maternity colonies, as observed in *P. austriacus* colonies, or roost near these colonies.

Significant fluctuations in the percentage of seropositive bats are indicative of several different episodes of EBLV-1 infection occurring in *P. austriacus* and *T. teniotis* colonies during the period of study. A quick increase and a high seropositive percentage after a lyssavirus episode are not unusual in a gregarious behavior species and could explain the sudden increase in the percentage of seropositive bats in *T. teniotis* and *P. austriacus* colonies. A similar quick increase with seropositive peaks of 60%–70% was observed in different colonies of *M. myotis* in Mallorca [23,24]. However, in *M. myotis* colonies, the evolution of seroprevalence after infection peaks follows a more gradual decline over subsequent years, until a new episode takes place, very different from what is observed here. The delay between the waves is then dependent on the rate of inflow of susceptible bats into the colonies as a consequence of new births, bat immigration from neighboring colonies and the expiration of EBLV-1-specific immunity in previously infected animals [23]. When a sufficient fraction of susceptible bats in the colony is reached, the virus spreads again if infected individuals join the colony. In the *T. teniotis* and *P. austriacus* colonies, the increase of seroprevalence is followed by a rapid decline until seropositive bats are not detected. The difference in the seropositive percentage evolution can be due to a higher rate of inflow of individuals in colonies of *T. teniotis* and *P. austriacus*. No data of inflow are available on *T. teniotis*, but very few recaptures were obtained during the study, indicating probably a high inflow rate in this colony. However, recapture rates in the *P. austriacus* colony were higher, suggesting a lower inflow in this species. Another hypothesis could be a different lifespan of immunity in these species. Recent studies estimated the lifespan of the *M. myotis* immunity from EBLV-1 to be around two years [36]. In this respect, it is possible that the immunity lifespan would be shorter in *P. austriacus* and *T. teniotis* than in *M. myotis*.

The best model obtained by GAM analysis indicated that inter-annual patterns of seroprevalence evolution were significantly different for *T. teniotis* and *P. austriacus*. Annual fluctuations could result from the behavioral ecology of the species involved [9]. *T. teniotis* and *P. austriacus* are two species with a different social organization and behavior. While *T. teniotis* forms large maternity colonies and can make long seasonal movements, *P. austriacus* forms smaller maternity colonies constituted by both sexes and makes shorter seasonal movements [37]. Different host ecology, behavior and movement could explain the different temporal variations in seroprevalence in these two species. Changes in density during migration or colony formation may affect contact rates and, thus, disease dynamics [9,38].

Differences in EBLV-1 exposure dynamics could also be related to host community composition and inter-species interaction. Higher EBLV-1 seroprevalence was observed in large and multispecies colonies compared to smaller and monospecific colonies, suggesting that interspecific virus transmission plays an important role in dynamics. A higher number of species might not only increase the rates of contact between bat groups, but could also facilitate virus entry or spread through the higher mobility of individuals among colonies, especially if there are migratory species [22]. In this sense, *M. schreibersii* (a species that often shares roost with *M. myotis*) has been considered as a regional reservoir and an essential species for EBLV-1 persistence in the Balearic Islands [36].

Other bat species present in the San Pedro pothole, such as *P. pipistrellus* and *P. kuhlii*, showed lower EBLV-1 seroprevalence than *P. austriacus* and *T. teniotis*. However, previous studies of bat rabies surveillance in Europe did not find EBLV-1-neutralizing antibodies in both species of *Pipistrellus* (for review see [39,40]). These results could be indicative of a low public health risk associated with these synanthropic species. Furthermore, the lack of a standardized serological test procedure, including arbitrary cut-off values, makes the comparison between previous European studies difficult. However, the higher values of EBLV-1 seroprevalence in our study could be due to differences in virus circulation and dynamics resulting from regional differences or selection of different types of colony (large multispecies maternity colonies in this case) [39,40]. Research programs that focus mainly on multi-host systems will help advance our understanding of the ecology of bat diseases.

## 5. Conclusions

This research addresses the role of multiple hosts in the infection dynamics of *Lyssavirus*. To advance our understanding of the ecology of bat lyssavirus, we report the results of specific EBLV-1 neutralizing antibody analysis in nine bat species roosting in the San Pedro de los Griegos pothole. These results suggest that most bats species roosted in this cave were exposed to the EBLV-1 lyssavirus. The evolution of seroprevalence in *T. teniotis* and *P. austriacus* colonies after infection peaks is different from that observed in *M. myotis* colonies. Differences in behavior ecology and population dynamics among bat species could explain the differences in the inter-annual variability of EBLV-1 seroprevalence.
